# Towards Integrated Youth Care: A Systematic Review of Facilitators and Barriers for Professionals

**DOI:** 10.1007/s10488-020-01049-8

**Published:** 2020-05-18

**Authors:** Laura A. Nooteboom, Eva A. Mulder, Chris H. Z. Kuiper, Olivier F. Colins, Robert R. J. M. Vermeiren

**Affiliations:** 1 Department of Child and Adolescent Psychiatry, Curium-Leiden University Medical Centre, Post Box 15, 2300 AA Leiden, The Netherlands; 2 Academic Workplace Youth at Risk, Pluryn, Nijmegen, The Netherlands; 3 Department of Child and Adolescent Psychiatry, Amsterdam University Medical Centre – Location VUMC, Amsterdam, The Netherlands; 4 Leiden University of Applied Sciences, Leiden, The Netherlands; 5 Horizon Youth Care and Special Education, Rotterdam, The Netherlands; 6 Department of Special Needs Education, Faculty of Psychology & Educational Sciences, Ghent University, Ghent, Belgium; 7 Youz, Parnassia Group, The Hague, The Netherlands

**Keywords:** Integrated care, Youth, Family, Mental health, Social care

## Abstract

To overcome fragmentation in support for children and their families with multiple and enduring problems across life domains, professionals increasingly try to organize integrated care. However, it is unclear what facilitators and barriers professionals experience when providing this integrated care. Our systematic review, including 55 studies from a broad variety of settings in Youth Care, showed that integrated care on a professional level is a multi-component entity consisting of several facilitators and barriers. Findings were clustered in seven general themes: ‘Child’s environment’, ‘Preconditions’, ‘Care process’, ‘Expertise’, ‘Interprofessional collaboration’, ‘Information exchange’, and ‘Professional identity’. The identified facilitators and barriers were generally consistent across studies, indicating broad applicability across settings and professional disciplines. This review clearly shows that when Youth Care professionals address a broad spectrum of problems, a variety of facilitators and barriers should be considered.

*Registration* PROSPERO, registration number CRD42018084527.

**Electronic supplementary material:**

The online version of this article (10.1007/s10488-020-01049-8) contains supplementary material, which is available to authorized users.

## Introduction

It is challenging for professionals in Youth Care to support children and their families with multiple and enduring problems across life domains (e.g., home, school, in the community; Tausendfreund et al. 2016). Although a small group, these children and their families experience a broad variety of problems, including psychosocial, emotional, cognitive and stress-related impairments, problems with alcohol and drugs, parental stress, child abuse, and socioeconomic disadvantages (Kolko and Perrin 2014; Tausendfreund et al. 2016). If left unaddressed, these problems can hinder normal child development and cause impairment that can endure into adulthood (Sellers et al. 2019). To timely and adequately address families’ needs, services in Youth Care encompass a wide range of support, including universal and preventive services, community centers, special education, specialized mental health care, child protection, social work, and residential treatment (Hilverdink 2013). However, the needs of families often exceed the expertise and possibilities of a single professional, service, or organization (Brooks et al. 2013). As a result, multiple professionals from a broad range of services with various expertise in Youth Care are involved in a family’s care process (e.g., psychiatrists, psychologists, primary care providers, family counselors, school counselors, and social workers).

Ideally, professionals in Youth Care collaboratively address multiple problems across life domains, while tailoring support to families’ needs (Hilverdink 2013; Krueger 2002). The number of professionals and type of professional expertise involved in a care process varies and depends on families’ needs. However, due to specific limitations in the access to services and fragmentation in terms of financing, there is often a mismatch between service delivery, professional culture, and the needs of families with multiple problems across life domains (Henderson et al. 2017; Kodner 2009). Consequently, professionals typically operate within their own specialty, while focusing on a restricted number of problems (Kodner 2009; Peek and The National Integration Academy Council 2013). A critical issue when focusing on a restricted number of problems is that the interrelatedness of the often co-occurring and exacerbating problems can be overlooked (Hawkins 2009; Tausendfreund et al. 2016). Moreover, a lack of coordination and collaboration in a care process can lead to fragmentation in support (Forman-Hoffman et al. 2017; Hawkins 2009; Tylee et al. 2007). Such fragmented care not only reduces client satisfaction and jeopardizes successful treatment outcomes (e.g., improved child and family functioning), it also increases service use and costs of Youth Care organizations (Kolko and Perrin 2014; Wissow et al. 2008).

To overcome fragmentation, there has been an increased focus on organizing integrated care in the last decade (WHO 2016). A problem with integrated care is its conceptual ambiguity: integrated care is organized in different ways and related to a broad variety of terms, including health services integration, care coordination, family-centered care, collaborative care, co-located care, and shared care (Armitage et al. 2009; Peek and The National Integration Academy Council 2013). Integrated care can refer to models, programs, collaborative agreements, working approaches, or specific interventions like case management, co-location, multidisciplinary care teams, and joint funding (WHO 2016). A common feature in models and terms is that integrated care seeks to improve quality of care for families by ensuring well-coordinated services around families’ needs by incorporating services, ensuring collaboration, and overcoming fragmentation (Kodner 2009; Wodchis et al. 2015). To ensure common understanding and improve conceptualization, we based our definition on three principal components of integrated care according to the WHO (2016): the delivery of coherent, coordinated, and continuous support, through different levels and sites within the care system (e.g., from universal services and primary care, through specialized mental health care centers), tailored to the needs of children and their families across several life domains.

Organizing integrated care has been deemed a complex and multi-component process. Integrated care can vary in intensity, spanning a continuum ranging from ad hoc linkage, over structured coordination, to full integration (Leutz 1999). Furthermore, organizing integrated care is more than forming networks, adding services, or providing multiple treatments alongside one another (Goodwin 2013). It requires processes on different complementary levels: organizational, clinical, and professional (Valentijn et al. 2013). The organizational level refers to relationships between services, coordinated policies, and activities to maintain networks. The clinical level refers to the primary process of care delivery to an individual: person-centered care in a single process across time, place, and discipline. The professional level refers to the delivery of integrated support: a professional’s behavior, attitudes, and expertise warranted to provide integrated care in collaboration with other professionals (Valentijn et al. 2013). Hence, integrated care on a professional level requires broad assessment of problems and needs, clear clinical pathways, and collaboration between professionals (Cooper et al. 2016; Kolko et al. 2014).

Previous reviews comparing models of integrated care have indicated that integrated care can improve the perceived quality of care and increase client satisfaction (Baxter et al. 2018; Cooper et al. 2016). However, evidence from these studies is mixed and emphasizes the importance of customized interventions or models to serve a specific population, setting, or context (Baxter et al. 2018; Patel et al. 2013). Various studies have sought to understand facilitators (i.e., components improving/enabling integrated care) and barriers (i.e., components limiting/obstructing integrated care) for professionals to integrated care in a specific context or to a specific population. For example, previous studies suggested that integrated care on a professional level requires timely identification of problems by means of adequate assessment of problems across life domains and monitoring progression during a care process (Bower and Gilbody 2005; Kolko et al. 2014), interprofessional collaboration (Cooper et al. 2016), and a flexibility to respond to the organizational differences across diverse settings (Ho et al. 2016). Other facilitators that were identified in general health care practice included clearly defined roles and responsibilities, a shared understanding of integrated care, and shared decision making on the intensity and type of support (Axelsson and Axelsson 2009; Cohen et al. 2015; Valentijn et al. 2013).

Notwithstanding that this previous research has furthered our understanding on aspects of integrated care, these studies were often conducted on a small-scale, limited to specific settings, or focused solely on one aspect of integrated care. Hence, the complexity of integrated care on a professional level remains understudied (Shaw et al. 2011; Sunderji, Waddell et al. 2016). Various scholars claimed that a deepened understanding of what professionals need to provide integrated care is essential to further improve support for children and their families (Richardson et al. 2017; Sunderji et al. 2017). Unfortunately, a systematic and comprehensive overview of facilitators and barriers for Youth Care professionals to provide integrated care has not been conducted yet. To fill this knowledge gap, the current systematic literature review aims to identify facilitators and barriers Youth Care professionals may encounter when providing integrated care across settings. A comprehensive review is of indisputable importance to formulate recommendations and guide Youth Care professionals and their organizations to organize and deliver integrated care (Grant and Booth 2009).

## Method

Our aim was to perform an extensive systematic literature review with rigorous analysis of facilitators and barriers for professionals to provide integrated care from a variety of settings, models, and populations seen in Youth Care. This approach was intentionally broad in order to find common understanding among different contexts, leading to facilitators and barriers that offer practical guidance across settings and professional disciplines. A research protocol to guide this review was prospectively registered in the International Database of Prospectively Registered Systematic Reviews in Health and Social Care (PROSPERO, registration number CRD42018084527). The Preferred Reporting Items for Systematic Reviews and Meta-Analysis (PRISMA) guidelines were followed to guide the review process and transparently report findings stemming from this review process (see Online Appendix A; Liberati et al. 2009). The literature review did not need approval from the Medic Ethics Review Committee (METC).

### Search Strategy

An extensive search strategy was designed in collaboration with an experienced medical research librarian from the Leiden University Medical Centre. Due to terminological variability, a set of search terms was formulated focusing on the following topics: integrated care, problems seen in Youth Care, and children/families. Search terms for integrated care included integrated care, family-centered care, co-located care, collaborative care, and shared care (Armitage et al. 2009; Peek and The National Integration Academy Council 2013). To account for the fact that Youth Care deals with families who display various (co-occurring) problems, we applied search terms referring to a broad variety of psychosocial, emotional, or cognitive problems, stress- and substance-related problems, socioeconomic disadvantages, and child abuse (Tausendfreund et al. 2016). To include a broad range of services in Youth Care, search terms encompassed child and youth (health) services, primary (health)care, child protective services, specialized mental health, and juvenile justice settings (Hilverdink 2013). To identify studies that focused on children and their families, we applied search terms such as child, pediatric, adolescents, families, and youth. To reduce the number of irrelevant studies, exclusion terms based on the eligibility criteria were added to the search strategy (e.g., internal medicine, elderly). Based on a preliminary screening, no potential relevant studies were missed when applying these exclusion terms. The detailed search strategy including the search terms can be found in Online Appendix B.

A computerized literature search was conducted in following electronic databases: PubMed, The Cochrane Library, Web of Science, Medline, and PsychINFO. The search was supplemented with literature obtained from the evidence-based Integrated Care Search from the International Foundation of Integrated Care (“Integrated Care Search”, no date). All identified studies were collected in the bibliographic reference manager Endnote®. Moreover, reference lists of studies selected for data extraction were screened for potential relevant publications that we might have missed during the computerized search.

### Eligibility Criteria

To be included, studies had to meet the following eligibility criteria:

- Focus on Youth Care: the support for children aged 0–18 and their families who experience a broad variety of problems across life domains, including psychosocial, emotional, cognitive and stress-related impairments, problems with alcohol and drugs, parental stress, child abuse, and socioeconomic disadvantages. Youth Care services included universal and preventive services, community centers, special education, specialized mental health care, child protection, social work, residential treatment, and juvenile justice settings.
- Respondents: professionals in Youth Care (YC practitioners), including psychiatrists, psychologists, pediatricians, primary care providers, social workers, family counselors, school counselors, and juvenile justice workers. Studies were also eligible for inclusion when they included a combination of Youth Care professionals and other respondents such as managers or parents.
- Focus on integrated care: any model, intervention, or working approach with a focus on overcoming fragmentation and promoting coherent support tailored to families’ needs. Integrated care includes the delivery of coherent, coordinated, and continuous support through different levels and sites within the care system, by increasing for example common cause, vision and strategy, joint funding or service delivery, and quality of support (Goodwin 2013; WHO 2016).
- Include outcomes as the result of an original study, review, or program evaluation, described as a facilitator (i.e., component identified as improving/enabling integrated care) or barrier (i.e., component identified as limiting/obstructing integrated care) for professionals.

Since research on integrated care comprises a variety of study designs spanning both quantitative and qualitative research methods, we aimed to include a broad range of original research articles (e.g., interviews, focus groups, case studies, action research, RCT’s, reviews). In that, we controlled for the source of evidence (e.g., whether the information came directly from professionals or other respondents) and paid specific attention to study quality by standardized quality appraisal. We searched for studies between January 1, 2002 and January 1, 2018 based on the increased focus on organizing integrated Youth Care services since the beginning of the 21th century (Shaw et al. 2011), Additionally, manuscripts had to be in English, peer-reviewed, and available as a full-text article.

To improve the transferability of results, non-western studies were excluded, since there are major differences in the organization of Youth Care across western and non-western cultures (Office of the Surgeon General Center for Mental Health Services 2001). Also, studies focusing on adults, solely on internal hospital settings, and publications such as conference abstracts or position papers were excluded from this review.

### Data Extraction and Synthesis

**Fig. 1:**
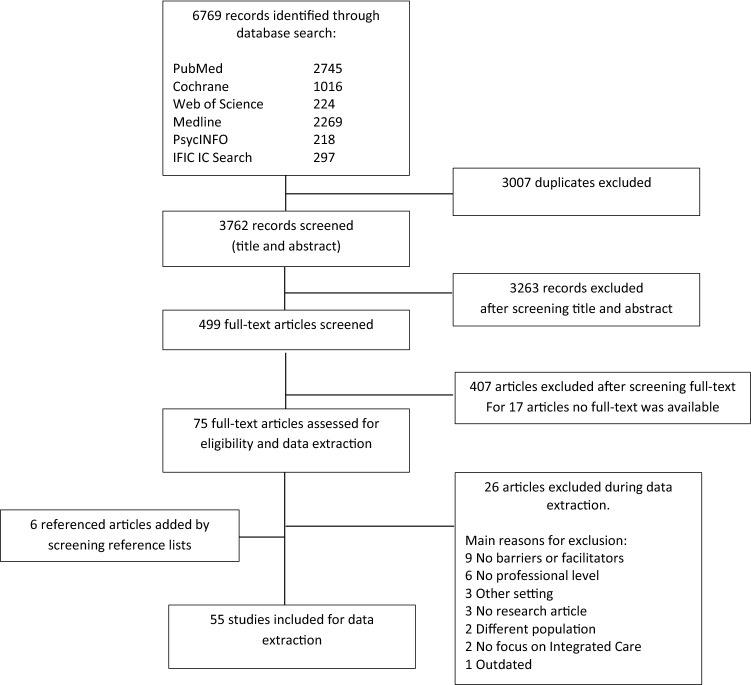
PRISMA flowchart

Study selection took place in several phases, summarized in a PRISMA flow diagram (see Fig. 1). Studies were independently reviewed by two researchers (LN and LK) based on the eligibility criteria. After studies were included, we derived first, second, and third order interpretations from the full-text manuscripts (Britten et al. 2002). The phases of data extraction and analysis were carefully prepared by the first author (LN) under supervision of two experienced qualitative researchers (CK and EM), by developing a standardized extraction form and plan for the thematic data synthesis. The first author extracted and analyzed the data, and three researchers (EM, CK, RV) verified data extraction, thematic analysis, and strength of evidence appraisal by several audit trails and reflexive meetings. Preliminary interpretation was discussed during these meetings to avoid bias.

All manuscripts were loaded in the qualitative data analysis software program Atlas.Ti (version 7). First-order interpretation was derived by means of open coding of the facilitators and barriers directly from the manuscript. Open coding is a common method in qualitative research and can be described as an interpretive process to gain new insights (Strauss and Corbin 1990). Open coding was conducted by conceptual labeling (coding) of identified fragments in the manuscripts and compare these fragments during further analysis. During the process of open coding, no additional codes were conceptualized for the last seven articles, indicating data saturation and completeness of our findings (Saunders et al. 2018). An a priori developed and pilot-tested standardized extraction form based on the Cochrane Data Extraction Template and the National Institute of Health and Clinical Excellence universal template (NICE) was used to register main outcomes from the open coding (facilitators and barriers); the second-order interpretation. This extraction form also included study characteristics (bibliographic information, aim, participants, study design, setting, and target population), source of evidence, a description of the integrated care process, and the level of integration (Leutz 1999). Furthermore, a third-order evaluation summary of the main outcomes was registered on the extraction form. For each study, the template was completed by the first author (LN) and verified by the research team (EM, RV and CK). The use of a standardized extraction template enabled us to register comparable information from each study. To avoid publication bias, all studies were controlled for repeated sample use. However, none of the included studies used repeated samples.

Thematic data synthesis was applied based on the open coding of facilitators and barriers. Using both inductive and deductive strategies, axial coding took place by analyzing and combining the coded fragments (Staa and Evers 2010). Facilitators and barriers were listed per theme to explore patterns in data and to create a conceptual model of themes and subthemes (Bearman and Dawson 2013). After summarizing these individual study outcomes, thematic descriptions were deductively compared with the initial study reports to limit possible adverse effects of prejudices and interpretation bias.

### Quality Appraisal

Quality of individual studies was critically appraised using standardized checklists developed by the Joanna Briggs Institute (2017). These checklists were available to assess a variety of study methods, including case reports, qualitative research, quasi-experimental studies, randomized controlled trials, and systematic reviews. With these forms, methodological quality of each study and possible bias in design, conduct, and analysis were rigorously appraised to inform synthesis and interpretation of the results. An objective ranking system was formulated in advance by the authors to assess the study quality based on the checklist. The quality ranking system included three categories: high ( more than 8 items checked), medium (6–8 items checked), or low quality (less than 6 items checked). An overview of study characteristics and critical appraisal scores can be found in Online Appendix C.

To assess strength of evidence of each subtheme, individual study outcomes were listed per subtheme. Critical appraisal was one of the main elements on which we based strength of evidence assessment. The first author labeled each facilitator and barrier with the quality label based on the critical appraisal (high, medium, or low). Then, to guide practice recommendations, strength of evidence was calculated for each subtheme by assessing (Harbour and Miller 2001; Ryan and Hill 2016):

- Quality of studies based on critical appraisal of individual studies: high (+; over 75% of the studies appraised as high quality), medium (±; 25–75% of the studies appraised as high quality), or low (−; under 25% of the studies appraised as high quality).
- ‘Size of evidence’: the number of studies within a subtheme. Since a golden standard for the number of studies was not available, size of evidence was based on a priori set standards: large (+; over 20 individual studies), medium (±; between 10 and 20 individual studies), or small (−; less than 10 individual studies).
- Context, categorized into global (+; a variety of studies from multiple contexts) and specific (−; all studies reported findings within the same specific context).
- Consistency of findings: assessed as consistent (+; all studies point to identical or similar conclusions), inconsistent (-; one or more studies directly refutes the findings of another study, in the same context or under the same conditions), or mixed (±; studies have produced results that contrast with those of other studies in different contexts or under different conditions).

Subsequently, strength of evidence was assessed based on the scores for each subscale, resulting in the following categories: very strong (++++), strong (+++), medium (++), limited (+), or no evidence (−). An overview of strength of evidence assessment for each subtheme can be found in Online Appendix D.

## Results

### Study Selection

Our database search identified 6.769 studies, resulting in 3.762 non-duplicate publications that were collected in the bibliographic reference manager (Endnote® X9). Study selection was conducted independently by two researchers (LN and LK) to reduce risk of bias and ascertain validity. Title and abstract were screened based on the eligibility criteria. In this round, we excluded studies solely focusing on medical conditions, adult populations, conference abstracts, position papers, and non-peer reviewed manuscripts. In case the two reviewers did not agree, the full-text was reviewed. In total, 499 studies were selected for full-text screening, leading to 75 studies eligible for data extraction. Main reasons for exclusion of these 424 articles were a lack of focus on professionals in Youth Care or integrated care (n = 129), lack of barriers or facilitators on a professional level (n = 127), no full-text available (n = 17), no research article (n = 87), different target population (n = 35), different setting (n = 29). The study selection inter-rater agreement as measured by Cohen’s Kappa was 0.70 for this round of inclusion, indicating substantial agreement between the two reviewers (Landis and Koch 1977). In four studies, disagreement was resolved through discussion and counselling by a third independent researcher (EM), who searched for consensus. In the other studies, reviewers solved their disagreement by collaboratively assessing the full-text articles. During the extraction phase, another 26 studies were excluded, mainly due to a lack of focus on facilitators or barriers on a professional level. After hand searching reference lists of the included studies, another 6 studies were eligible for inclusion. In total, 55 studies were included in this review.

### Study Characteristics

Of the 55 included studies selected within the span of 2002–2018, more than half (n = 33; 60%) were published after 2011. The included studies covered multiple settings in Youth Care. Specifically, all studies took place in primary care (n = 33) or in specialized mental health care settings (n = 22), in combination with for example educational (n = 6), child welfare (n = 3), juvenile justice (n = 4), substance abuse treatment (n = 2), or child protection (n = 3) settings. Most studies focused on mental health problems of children (n = 32), often in combination with child maltreatment, substance abuse, and psychosocial support of family members. Integrated care models and approaches varied widely across studies, and the level of integration spanned a continuum ranging from ad hoc linkage, over structured coordination to full integration (Leutz 1999). Examples of integrated care models or approaches included in our study sample were collaborative screening, care coordination, shared referral, service networks, collaborative training, multidisciplinary teams, and co-location.

In 43 studies, Youth Care professionals were the primary respondents, including psychologists, parent support workers, child psychiatrists, pediatric nurses, social workers, special education workers, and primary care providers. Study methodology varied across studies, including questionnaires, interviews, focus groups, observations, literature reviews, case descriptions, action research, or a combination of these methods. Based on critical appraisal of individual studies, 30 studies were appraised of high quality (e.g., based on clear and comprehensive report of research methodology), 7 studies of medium quality, and 18 studies of low quality. The low-quality studies were often small-scale program evaluations, lacking a clear design or reported methodology. A complete overview of individual study characteristics and the critical appraisal can be found in Online Appendix C.

### Outcomes

**Fig. 2:**
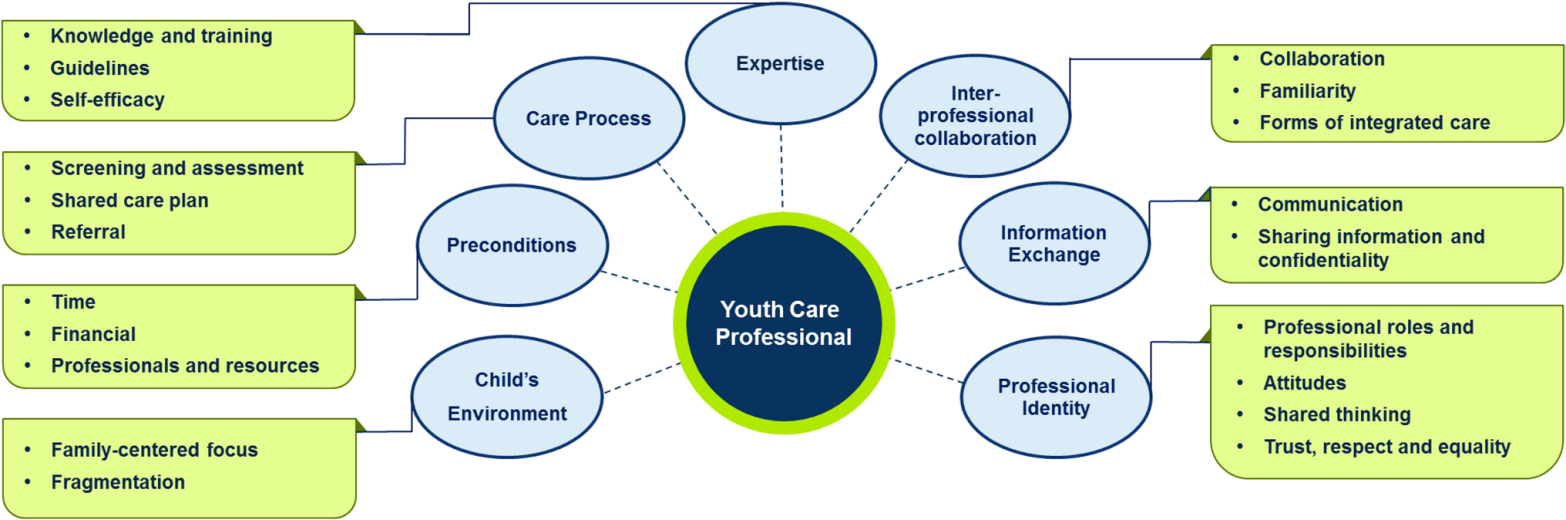
Thematic overview of facilitators and barriers for Youth Care professionals to provide integrated care

The aim of this review was to identify facilitators and barriers for professionals to provide integrated care. Since the identified facilitators (e.g., sufficient time) were often the opposite of barriers (e.g., lack of time) and vice versa, we chose for a thematic clustering of facilitators and barriers that were identified during the open coding. The thematic clustering resulted in seven overarching themes and 24 subthemes (see Table 1 for a description of each subtheme, Fig. 2 for an overview of themes and subthemes). The coded facilitators and barriers were listed to explore patterns by means of axial coding, leading to a conceptual model of subthemes (Bearman and Dawson 2013). The conceptual model circulated in the research team for verification. The final themes and subthemes were formulated during reflexive meetings (LN, EM, CK, RV). This approach led to a variety of (interrelated) themes that offer practical guidance for professionals to provide integrated care. Strength of evidence was rated for each subtheme based on our rating scheme and varied from medium to very strong. This is an indication that all subthemes can be interpreted with confidence. Most subthemes included a high number of studies with medium quality. In all subthemes, the context was assessed as ‘general’. Sixteen subthemes were rated as ‘consistent’, the other eight were ‘mixed’, indicating that the subthemes are applicable for professionals in a variety of settings in Youth Care. Detailed findings of strength of evidence appraisal and presence of individual studies within each subtheme are listed in Online Appendix D. To improve readability, studies presented in the result section received a study number.

**Table 1:** Themes and subthemes based on barriers and facilitators

Theme	Subthemes	Subtheme description	Number of studies	Strength of evidence
Child’s environment	Family-centered focus	A holistic approach on a family's welfare	17	Medium—strong
	Fragmentation	Collaboration between education and health care systems	5	Strong
Preconditions	Time	Time to address a broad spectrum of problems and for interprofessional collaboration	25	Strong—very strong
	Financial	Financial support and funding streams	7	Strong
	Professionals and resources	Availability of professionals and services	28	Strong
Care Process	Screening and assessment	Broad assessment of problems and the use of screening tools	21	Strong
	Shared Care plan	Several perspectives and goals in a comprehensive care plan	5	Medium—strong
	Referral	Transition between care providers	9	Medium—strong
Expertise	Knowledge and training	Extending knowledge by means of training	37	Strong
	Guidelines	The use of evidence-based guidelines to support professionals	13	Strong
	Self-efficacy	Confidence and comfort of professionals to provide integrated care	15	Strong
Interprofessional collaboration	General aspects of collaboration	The importance of interprofessional relationships	10	Medium—strong
	Familiarity with other professionals	Knowing and understanding other professionals’ expertise	16	Strong—very strong
	Forms			
	Co-location	Multiple services at one location	19	Strong
	Multidisciplinary meetings	Meetings where professionals share knowledge, highlight concerns and reflect on care processes	13	Strong—very strong
	Consultation	Consultation of other (specialist) professionals	18	Strong
	Care coordination	Professional with the specific task to coordinate a care process	6	Medium
Information exchange	Communication	A shared language and motivation to communicate	22	Strong—very strong
	Sharing information and confidentiality	Content and frequency of information exchange, shared medical records and legal guidelines for sharing information	27	Strong
Professional identity	Professional roles and responsibilities	Clarity and expectations about professional roles, sharing responsibility	27	Strong
	Attitudes	Attitudes and commitment towards integrated care and collaboration	16	Strong
	Shared thinking	A shared foundation in thoughts, aims, priorities, and values	22	Strong—very strong
	Trust, respect and equality	Mutual trust, respect for other professionals and perceived equality	20	Strong

### Theme 1: Child’s Environment

The theme ‘Child’s environment’ was divided into two subthemes with barriers and facilitators: family-centered focus (17 studies) and fragmentation (5 studies).

#### Family-Centered Focus

A holistic focus with both a generalist view on the entire family's welfare and a specific focus on individual needs was reported as a facilitator in nine studies (6, 11, 22, 29, 34, 42, 47, 49, 50). To accomplish a balance between a generalist view and a specialist approach of problems, professionals should be able to accurately prioritize problems and decide on the focus of support when considering different life domains (22, 32). Other reported facilitators were being aware of the other professionals’ context and being able to respond competently to various situations (44, 45, 54).

A reported barrier for professionals was to maintain a holistic focus while at the same time prioritize problems, especially for children with severe problems (25, 51). Studies suggested that the feasibility of combining a specialist and generalist approach was complicated by the unpredictable and episodic nature of problems, incompatible needs of multiple family members, or concerns about a child’s safety (22, 53). Other reported barriers were differences in perspectives on the primary client within one family, and the perception that other professionals solely pay attention to their own individual client or field of expertise (11, 53, 54).

#### Fragmentation

The gap in collaboration between professionals working in the educational system (e.g., teachers) and professionals from other settings in Youth Care was reported as a major barrier in various studies (8, 11, 23, 36, 39). These studies suggested that differences in focus, culture, and procedures lead to disconnection and fragmentation between the two systems, hampering Youth Care professionals to provide integrated care.

### Theme 2: Preconditions

Facilitators and barriers of the theme ‘Preconditions’ were described in three subthemes: time (25 studies), financial (7 studies), and professionals and resources (28 studies).

#### Time

Reported facilitators were flexible schedules, sufficient time for interprofessional team development, reflection on collaboration, and clinical discussions (10, 22, 37, 39, 45, 47, 49). On the other hand, a lack of time during regular visits to address a broad spectrum of problems was reported as a major barrier (5, 8, 17, 27, 36, 39, 42, 45, 46, 49). Also, interprofessional collaboration was described as time consuming (22, 24, 35, 37, 45, 47), with inflexible schedules of professionals, a lack of time for communication, and leaving collaboration to chance as reported barriers (2, 12, 19, 21, 23, 51, 52, 54, 55).

#### Financial

A lack of financial support for collaborative activities, separate funding streams, and differences in reimbursement rates for various health codes or diagnoses were reported barriers for professionals (2, 5, 21, 33, 39, 42, 47).

#### Professionals and Resources

Reported facilitators were the availability of professionals and adequate resources such as specific intervention programs (2, 7, 48, 50). Hiring additional staff was also described as a facilitator, under the condition that new staff has a notably distinct role or expertise (1, 2, 3, 7, 27, 28, 41, 46). Estimating the adequate number of professionals needed to provide integrated care was stressed as complex, due to the fluctuating demands and specific needs of families at various times (2, 39, 53). Reported barriers in availability of professionals were related to frequent turnover of professionals (24), high clinical demands (33), and a lack of transparency in the availability of services (39, 51, 54). Other barriers included specific demands of services (i.e., a focus on single problems that caused refusal of children and families with interrelated problems) and a shortage of trained professionals for assessment, treatment, or care coordination (1, 6, 13, 19, 32, 49, 52). Also, the lack of availability of specialist services was identified as a barrier, often leading to long waiting lists and gaps in service provision (9, 11, 17, 24, 29, 39, 50).

### Theme 3: Care Process

This theme was divided into three general aspects of care processes in Youth Care: broad assessment and the use of screening tools (21 studies), the use of a shared care plan (5 studies), and the referral process (i.e., the transition between care providers; 9 studies).

#### Screening and Assessment

Reported facilitators for broad screening and assessment were joint assessment (i.e., professionals with supplementary expertise jointly assess children and families; 50) and the use of validated screening tools to identify risks and strengths across multiple life domains (1, 8, 12, 15, 17, 26, 27, 28, 29, 32, 38, 41, 46, 49). Screening tools deemed important in multiple studies, because they seemed to increase the capacity and confidence of professionals to assessing a broad spectrum of problems (35), discussing strengths and weaknesses with families (51), and sorting out diagnostic criteria and comorbidities (17). However, the following barriers to the implementation of screening tools were identified: difficulties in (timely) application of tools, interpretation of test results, formulating a follow-up plan based on the screening results, and reporting the screening results to families (11, 17, 21, 27, 33, 41, 49, 52).

#### Shared Care Plan

Five studies reported a shared care plan as a facilitator: a mutually understood and agreed upon care plan, including an overview of a families’ needs and goals (7, 25, 38, 39, 50). The plan should be flexible and adjustable to the needs of families at any time.

#### Referral

Identified facilitators in the referral process (i.e., the transition between care providers) were: clear referral pathways, warm handoffs between professionals, and shared intervention planning (2, 13, 29, 38, 41, 52). On the contrary, reported barriers were a lack of sharing information and miscommunication between professionals at transition points, leading to a discontinuity of care (24, 50, 51).

### Theme 4: Expertise

The theme ‘Expertise’ was divided into three subthemes with barriers and facilitators, that were often mentioned in relation to each other: knowledge and training (37 studies), the use of guidelines (13 studies), and self-efficacy (15 studies).

#### Knowledge and Training

A broad range of knowledge concerning problems seen in Youth Care was a reported facilitator for professionals (21, 44). Multiple studies indicated that training expands knowledge of this broad range of problems, resulting in improved self-efficacy of professionals to provide integrated care (5, 13, 18, 20). Also, (joint) training in interprofessional collaboration was a reported facilitator (16, 17, 18, 20, 25, 29, 30, 33, 41, 50), described in several forms: multidisciplinary training, working alongside a professional with different expertise, and interdisciplinary education curricula (2, 4, 10, 14, 19, 30, 32, 35, 38, 46). Studies suggested that study material should be available after training to keep knowledge up to date (25, 39, 49).

A frequent reported barrier was a professional’s lack of knowledge, for example regarding triaging and referring to other services (1, 4, 5, 11, 15, 18, 21, 24, 25, 27, 46, 51, 53, 54). Also, studies yielded mixed evidence on the objectives of training. In fact, it remains unclear whether the focus of training should be on enhancing broad knowledge of a spectrum of problems (1, 5, 11, 24, 26, 32, 38, 46, 52), or on enhancing elaborated knowledge of specific problems (10, 12, 15, 18, 27, 35, 54). Also, findings concerning whether training should be on the job were inconsistent (35, 41, 46). Professionals can experience difficulties in prioritizing training due to high work demands, a lack of time, or little motivation (3, 17, 25). Moreover, evidence regarding the effect of training on a professional’s self-efficacy was inconsistent: one study described that despite training, professionals still experienced a lack of knowledge and confidence to provide integrated care (39).

#### Guidelines

A reported facilitator was the presence of evidence-based practice guidelines or protocols for interprofessional collaboration (3, 7, 8, 19, 23, 25, 27, 30, 37, 38, 39, 42, 50). These reported guidelines supported professionals in the recognition and treatment of problems, and in interprofessional collaboration by describing standardized processes for sharing information, decision making, and treatment planning.

#### Self-Efficacy

Feeling comfortable and competent (i.e., self-efficacy) to assess a broad spectrum of problems and collaborate with various professionals was often mentioned as a facilitator in relation to a professional’s knowledge (9, 17, 20, 30, 49, 53). Self-efficacy was found to be improved by a professional’s perception of empowerment (i.e., the validity to act and the feeling of control over their work), and positive feedback from families (17, 45). Reported barriers were interprofessional challenges and addressing a broad spectrum of severe problems, driving professionals out of their comfort zone and thereby leading to a lack of self-efficacy (9, 15, 17, 20, 24, 27, 29, 33, 35, 51).

### Theme 5: Interprofessional Collaboration

Facilitators and barriers of the theme ‘Interprofessional collaboration’ (i.e., working across organizational and professional boundaries) were described in three subthemes: general aspects of interprofessional collaboration (10 studies), familiarity with other professionals (16 studies), and various forms of interprofessional collaboration (19 studies on co-location, 13 on multidisciplinary meetings, 18 on consultation, and 6 on care coordination).

#### General Aspects of Collaboration

Reported facilitators to collaboration were concrete objectives and conditions for collaboration, timely involvement of other professionals during early stages of care, and sharing information. Other facilitators were investing in team development and the creating of supportive relationships with other professionals that are based on mutual respect (3, 22, 29, 34, 39, 40, 42, 45). Studies indicated that both structural collaboration in fully integrated care teams, and flexible collaboration on a case level can facilitate integrated care (19, 29). When forming these multidisciplinary care teams, it is important to be aware of the size of a care team: involving too many professionals was described as a barrier (37, 39).

#### Familiarity with Other Professionals

Familiarity with other professionals was reported as a facilitator, by adequately incorporating different perspectives, and understanding other professionals’ contributions and day-to-day practice (3, 6, 11, 12, 23, 32, 33, 37, 42, 46, 50, 53). Familiarization can be improved by sharing brief bibliographical information, evaluate strengths or limitations in collaboration, and regular clinical case discussions (12, 14, 23, 53). Being unfamiliar with other professionals’ care systems, services, language, and protocols were reported barriers that led to frustration and underutilization of services (22, 29, 33, 37, 45, 50).

#### Forms of Integrated Care

Co-location and multidisciplinary meetings seemed to broaden the scope of care provided, increase information exchange, and improve opportunities for learning (6, 16, 19, 21, 33, 37, 39, 46, 47, 48, 50, 52, 53). Also, co-location and multidisciplinary meetings were described as leading to more frequent contact moments and warm handoffs (4, 10, 28, 29, 41, 42, 52), positive perception of interprofessional collaboration (16, 43), more appropriate assessment or referral (22, 31, 33), and eventually time saving (30). Consultation of other professionals was a reported facilitator that led to a feeling of support, improved staff wellbeing, and increased self-efficacy in supporting families (1, 7, 10, 12, 15, 17, 22, 29, 32, 38, 41, 50, 52). A care coordinator was described as a facilitator to integrated care by stimulating interprofessional communication, and having a complete overview of families’ needs and the availability of support (7, 10, 29, 42, 50, 55). Although all forms of integrated care were reported as facilitators, one study pointed out that it is not necessarily the physical proximity of professionals that influences integrated care, but the level of communication (23). Reported barriers concerning various forms of integrated care were a shortage of specialized professionals available for consultation or to work at co-located sites (15, 35, 51), a shortage of time and workspace (16, 21), and inflexible schedules of professionals to participate in meetings (33, 48). Other barriers were a lack of structure or coordination during multidisciplinary meetings (48) and a lack of support and financial compensation for consultation activities (20, 24, 29, 40, 50).

### Theme 6: Information Exchange

This theme was strongly related to the theme ‘Interprofessional collaboration’, as it is about the frequency and consent of sharing information between professionals. The theme ‘Information exchange’ was divided into two subthemes: communication (22 studies), and sharing information and confidentiality (27 studies).

#### Communication

Reported facilitators were clear and transparent communication between professionals (9, 27, 32, 38, 50, 53). Specifically, a shared language, being available for contact, electronic reminders for communication, and acknowledging the importance of clear and transparent communication, facilitated clear and transparent communication (6, 12, 23, 24, 30, 37, 38, 39, 45, 53). Other facilitators were: collaboratively defining expectations for the content, frequency and timing of communication, evaluation of communication processes, understanding differences in communication styles, and effective oral and written communication skills (9, 12, 23, 26, 34, 38, 42, 46, 48). Reported barriers in communication included a perceived unavailability or unwillingness to communicate, inadequate timing, a lack of reciprocity, and a lack of shared terminology (9, 11, 25, 36, 42, 44, 50, 53).

#### Sharing Information and Confidentiality

Sharing accessible and comprehensible information with other professionals was reported as leading to role expansion and shared knowledge, both facilitators to integrated care (19, 26, 28). Also, shared medical records (e.g., bidirectional system for sharing information, advice, and feedback) were identified as facilitators, by reducing service duplication, improving regular communication and shared understanding of families’ needs (9, 12, 14, 21, 23, 27, 30, 32, 33, 36, 38, 41, 47, 48, 51). Professionals’ perception that their input contributed to a care process was deemed important in sharing information (16). Also, discussing the importance of sharing information or possible confidentiality issues with families was also described as a facilitator (38, 46, 47). Reported barriers were a lack of information exchange, unawareness of the content of information that other professionals needed, and a failure to understand the provided information (16, 23, 29, 33, 34, 53). Also, misunderstanding of confidentiality requirements across disciplines was a barrier for professionals in sharing information (21, 29, 32, 37, 38, 42, 46, 50, 54).

### Theme 7: Professional Identity

Facilitators and barriers of the theme ‘Professional identity’ were described in four subthemes: professional roles and responsibilities (27 studies), attitudes (16 studies), shared thinking (22 studies), and trust, respect and equity (20 studies).

#### Professional Roles and Responsibilities

Clear professional roles, realistic expectations of other professionals, and being aware of professionals’ own boundaries and responsibilities were identified as facilitators (14, 21, 22, 26, 29, 30, 38, 42, 48, 53). Other facilitators were being able to recognize and take responsibility during a care process (45), and the feeling of shared responsibility over complex cases (29, 30, 33, 34, 37). Some studies reported that roles and responsibilities should be discussed and set in advance (29, 41). Yet, other studies described flexible roles and responsibilities as facilitators to integrated care, enabling professionals to response to the changing needs of families (19, 22, 45, 53). Reported barriers were unclear or competing roles and unrealistic expectations of other professionals, that often led to confusion and conflicts among professionals (6, 11, 22, 23, 29, 36, 37, 39, 42, 44, 45, 50, 53, 54, 55). Other barriers were disagreement over responsibilities, confusion about legal liability, and a perceived lack of reciprocity in collaboration, leading to different feelings of ownership, unclear allocation of tasks, and finger-pointing (6, 24, 29, 48, 50, 51, 54, 55).

#### Attitudes

Reported facilitators were positive attitudes and commitment towards integrated care or interprofessional collaboration (12, 17, 19, 22, 23, 24, 29, 44, 45, 55). In contrast, reported barriers were a lack of commitment, lack of appreciation of other professionals, and negative experiences with collaboration (4, 14, 17, 19, 22, 23, 33, 34, 42, 54).

#### Shared Thinking

Reported facilitators were integrating viewpoints of other professionals in comprehensive care plans (38, 53) and a shared foundation in thoughts, values, knowledge, and working styles (3, 12, 14, 26, 30, 40, 45, 47). Reported barriers were competing work demands, differences in priorities, various explanatory models, and different (hierarchical) relations between professionals and families (6, 9, 11, 14, 19, 25, 34, 37, 40, 42, 50, 52, 53, 54, 55).

#### Trust, Respect and Equality

Mutual trust, respect, appreciation of the diversity of professional backgrounds, and equity between professionals were found to facilitate integrated care (6, 19, 26, 29, 35, 37, 38, 42, 44, 45, 47, 50, 54). Reported barriers included a lack of trust and respect, perceived inequality between professionals, concerns about confidentiality, and a lack of commonality in the approach of families and other professionals (11, 16, 19, 24, 29, 33, 34, 40, 44, 45, 48, 50, 54).

## Discussion

In this systematic review, we aimed to identify facilitators and barriers for professionals to provide integrated care from a broad variety of studies. We included studies with diverse methodologies, populations, settings in Youth Care, and types of integrated care to find common understanding among different contexts and professional disciplines. The current review identified seven themes and 24 subthemes of barriers and facilitators for Youth Care professionals to provide integrated care. Despite the diversity in studies included, the strength of evidence rating showed that the barriers and facilitators were generally consistent across studies and thereby applicable in a variety of settings.

Overall, the broad variety of facilitators and barriers clearly shows that providing integrated care is a multicomponent and complex process. An important aspect of integrated care is that it is not limited to, or focused on one specific setting or individual, but that it is provided throughout the entire continuum of care. Whether professionals work in universal services or specialized mental health centers, integrated care is influenced by multiple facilitators and barriers on a professional level that require interprofessional collaboration and the addressing of a broad variety of problems. As described in previous research (Curry and Ham 2010), the variety of studies and integrated care approaches suggest that there is no single approach or model to integrated care that can be applied universally. Hence, different approaches might be needed to fit local and individual needs.

Reflecting upon the themes and subthemes, we conclude that facilitators and barriers regarding interprofessional collaboration were most frequently reported (e.g., time for interprofessional team development, training in interprofessional collaboration, several forms of collaboration, sharing information with other professionals). This finding is consistent with prior work that studied integrated care for children and adolescents with mental health problems (Cooper et al 2016; Richardson et al. 2017). In addition, findings reported in the themes ‘Child’s environment’, ‘Care process’ and ‘Expertise’ suggest that broad assessment of problems and timely identification of the intensity and type of care a family needs are other important aspects of integrated care.

Echoing prior work, our review indicates that the organization of integrated care is substantially influenced by processes on a professional level (Goodwin 2013; Valentijn et al. 2013). We suggest that when further developing the concept of integrated care, the focus should be on the professionals involved in integrated care on a day-to-day-basis, instead of solely considering interprofessional collaboration at organizational level (Stein 2016; World Health Organization 2016). In the following section, we reflect upon our findings in depth and formulate implications for practice, education, and further research.

### Specialist Versus Generalist Approach

Various studies emphasized the importance of expanding knowledge and skills of Youth Care professionals. Echoing prior recommendations (Sunderji et al. 2016), there is a need for role changes and advanced competences for professionals in attaining both a generalist view of a family's welfare, and a specialist’s approach on specific needs of each individual family member. However, studies that focused on the knowledge professionals should possess yielded mixed findings (see Theme 1 ‘Child’s environment’ and Theme 4 ‘Expertise’). Specifically, it remains unclear whether this knowledge should be broad (generalist), in depth (specialist), or a combination of both. Although the importance of diverse knowledge can be inherent to the broad spectrum of problems seen in Youth Care, it seems unrealistic that one individual professional can learn and apply all available knowledge in its day-to-day practice. As long as there is no consensus on the basic knowledge and skills a Youth Care professional should possess, it remains unclear whether expanding professionals’ knowledge facilitates integrated care (Armitage et al. 2009; Kodner 2009). Moreover, previous research suggested that working in multidisciplinary teams can expand the scope of care provided when supporting families in Youth Care (Anderson-Butcher et al. 2002; Golding 2010; Nolan et al. 2016). To efficiently compose these multidisciplinary teams, we strongly recommend to further examine what disciplines, knowledge, and skills are needed in a multidisciplinary team to provide integrated support in Youth Care.

Working alongside a professional with different expertise and collaboratively reflecting on multidisciplinary care processes, can expand a professional’s knowledge and skills (see Theme 4, ‘Expertise’). Future studies must examine the effectiveness of several forms of interprofessional learning in integrated care. For example, previous studies suggested that active involvement in a continuous learning cycle with a focus on improving professionals’ competences, interprofessional team development, and clinical case discussions facilitates professionals in expanding their knowledge and skills (Langins and Borgermans 2015; Stein 2016). When developing learning methods for interprofessional collaboration in Youth Care, the high work demands and difficulties in prioritizing learning activities should be considered. Therefore, we recommend to engage professionals in collaboratively developing learning methods, since this might lead to increased applicability and validity in practice.

### Assessment and Prioritizing of Problems

Broad assessment of problems and timely identification of the intensity and type of care a family needs are important aspects of integrated care (see Theme 1 ‘Child’s environment’, Theme 3 ‘Care process’, and Theme 4 ‘Expertise’). Yet, issues that emerged when reflecting upon these themes were difficulties in prioritizing problems, leading to problems in determining the focus of support. These difficulties seemed related to the interaction of problems within one individual or between different family members. Specifically, the needs of family members can conflict, and professionals can have different perceptions about the primary client within one family. Also, previous research stated that professionals can experience difficulties in incorporating clients’ viewpoints in decision-making processes (Simmons et al. 2018). To enhance professionals’ skills in prioritizing problems and shared decision making, we recommend to frequently discuss priorities with families and thereby incorporate their perspectives in the care process. Moreover, our findings in the subtheme ‘Guidelines’ support the recommendation of the World Health Organization (2016), namely that the use of practice-based guidelines facilitates professionals in prioritizing and decision-making processes. However, details on the implementation and effectiveness of evidence-based practice guidelines were not reported in the studies included in this review. As we know from previous research, adherence to guidelines in applied settings improves when paying specific attention to a structured and tailored implementation in collaboration with the end-users (Fisher et al. 2016).

### Professional Roles and Responsibilities

It is often difficult for professionals to define clear roles in interprofessional collaboration and to share responsibility over a care process (Cooper et al. 2016). Studies in this review indicated that clear roles and responsibilities that are set in advance facilitate interprofessional collaboration (see Theme 7 ‘Professional identity’). However, other studies reported that roles and responsibilities must be flexible when responding to the changing needs of families in Youth Care. This apparent inconsistency (e.g., fixed versus flexible roles) can be attributed to the variety of professional disciplines involved in care processes and the different needs across families. In line with previous research (Valentijn et al. 2013), we suggest that it is crucial to continuously evaluate roles and responsibilities during a care process, with all stakeholders involved. Yet, it remains unclear how and how often professionals should hold these evaluative meetings. Also, previous research reported a lack of structure during these meetings as a barrier (see Theme 5 ‘Interprofessional Collaboration’). Hence, to guide professionals in organizing these evaluative meetings, future research should study the effectiveness of various forms of evaluative meetings in practice, for example by means of action-research.

### Time to Invest in Integrated Care

Supporting families with various needs and interprofessional collaboration are time-consuming processes (see Theme 2 ‘Preconditions’). Based on the reviewed studies, we suggest that when trying to optimize integrated care processes and eventually save time, it is necessary to invest in prolonged visit times, time for interprofessional team development, and evaluative meetings. However, since a lack of time is a well-known problem in Youth Care, investing time in interprofessional team development and case discussions is limited. Therefore, it is important that professionals are supported in effectively organizing and prioritizing these activities, for example by their management or by practice-based guidelines.

Additionally, it is challenging to estimate the amount of time and number of professionals that are needed in a single care process (see Theme 2 ‘Preconditions’). For example, needs differ between families, and fluctuate over time within a family. As we already suggested, more work needs to be done in determining patterns in families’ needs, to establish a better estimation of the required time, disciplines and number of professionals. We also recommend examining the long-term effects of integrated care by setting up a continuous routine monitoring system (see also: Tsiachristas et al. 2016). Such a system could, for example, track families’ needs and goal attainment, service utilization and costs of integrated care.

### Attitudes, Skills and Competences

Providing integrated care requires specific attitudes, skills, and competences of professionals, including: (i) positive attitudes and commitment of Youth Care professionals towards integrated care and interprofessional collaboration, (ii) the ability to incorporate viewpoints of several professionals into a comprehensive care plan, and (iii) acknowledgement of the importance of communication and effective communication skills. Previous research demonstrated that it is not necessarily the physical proximity of professionals, but the level of communication that influences integrated care (Greene et al. 2016). This indicates that interprofessional communication skills are important to consider when organizing integrated care and must be part of training and education programs for (future) professionals.

Moreover, multiple studies in our review showed that professionals in Youth Care should be able to timely and adequately estimate when and what additional expertise is needed in a care process (see Theme 2 ‘Preconditions’ and Theme 4 ‘Expertise’). Although this was beyond the scope of our review, we suggest that there might be differences in professionals’ perspectives on what expertise is needed, at what time, and to what extent. This is an important issue for future research, since there is often a broad variety of professional disciplines involved in a care process. We recommend the use of qualitative research methods to examine what professionals need in deciding the focus of support and the expertise required to tailor support to families’ needs.

### Strengths and Limitations

This review has several strengths. First, by prospectively registering our review protocol in PROSPERO we kept track of any unexpected differences during the review process that, fortunately, did not occur. Thereby we reduced the risk of reporting bias. Second, our review covered relevant literature regarding facilitators and barriers for Youth Care professionals, due to our extensive search strategy and rigorous analysis. Third, to increase the applicability and generalizability of the results, we included studies of a broad range of settings within the field of Youth Care (i.e., mental health care, primary care, education, child welfare, juvenile justice, substance abuse settings, and child protection). The consistency of reported facilitators and barriers across settings indicate broad applicability across settings and professional disciplines.

Of course, our results should be interpreted in the context of various limitations. Since there was no common approach to measure outcomes across studies, it was difficult to provide an overall comparative analysis of the impact of barriers and facilitators identified in the studies. By means of an a priori developed and pilot tested standardized extraction form, we registered main outcomes for each included study, a working approach that facilitated the collection of comparable information (Burau 2012). Studies were analyzed by means of open coding, followed by axial coding to explore patterns in coded fragments (Strauss and Corbin 1990). Data saturation was reached when coding the results, an indication that our review provides an extensive overview of facilitators and barriers from existing literature. Due to the conceptual ambiguity of integrated care (Armitage et al. 2009; Peek and The National Integration Academy Council 2013), our search terms were broadly defined. However, the definition of integrated care slightly differed across the included articles. We intended to control for these differences by rating the intensity of integrated care and extracting a description of integrated care directly from the included studies on a standardized extraction form. Moreover, we limited our search to English, peer-reviewed articles, with both qualitative and quantitative research designs and program evaluations. Adversely, we might have missed some relevant information from reports or other gray literature.

We intended to control for quality by critically appraising the quality of individual studies and assessing the strength of evidence per subtheme. However, we did include 18 studies of low quality, for example studies with uncontrolled or unclear designs, and small or unclear samples. We aimed to control for these low-quality studies by including quality of studies in our strength of evidence appraisal. Most of the included studies did not report any effect sizes, hence it was not possible to estimate to what extend facilitators and barriers affected practice. Likewise, the study design did not allow to scrutinize if the distilled themes interacted with each other. As a result, barriers and facilitators are separated in themes that might be interrelated. These limitations have been mentioned in previous reviews in the field of integrated care (Cooper et al. 2016; Richardson et al. 2017), stressing that there is a need for high quality studies to the effects of integrated care in practice (e.g., randomized controlled trials). However, since integrated care is such a context-dependent and multi-component process on several levels, conducting a randomized controlled trial is challenging. In line with previous research (Wisdom et al. 2012), we therefore suggest that mixed method research, using both quantitative and qualitative research methods is needed to further our understanding of integrated care on a professional level.

## Conclusion

Overall, this review clearly shows that providing integrated care is a multi-component and complex process, hallmarked by various facilitators and barriers for professionals. With our review, it was possible to identify barriers and facilitators that were generally consistent from a variety of studies, indicating broad applicability across settings and professional disciplines in Youth Care. The identified barriers and facilitators were related to interprofessional collaboration, including various forms of interprofessional collaboration, efficient information exchange, flexible professional roles, and sharing responsibilities. We also identified facilitators and barriers for professionals in the assessment of a broad spectrum of problems, timely identification of problems, and prioritizing the needs of families.

Currently, the major focus when organizing integrated care is at an organizational level (Goodwin 2013). This review demonstrated that considering various aspects of integrated care on a professional level is critical to organize integrated care in practice. Moreover, in education and training for (future) professionals, attention should be paid to various aspects of integrated care like interprofessional communication, the application of practice-based guidelines, and evaluation and reflection on roles and responsibilities. Importantly, a consensus on the general knowledge and skills Youth Care professionals should possess, and disciplines that should be involved in a care process are needed to improve integrated care in practice and develop curriculum methods for future professionals in Youth Care.

## Electronic supplementary material

Below is the link to the electronic supplementary material.

Supplementary file1 (DOC 61 kb)

Supplementary file2 (DOCX 14 kb)

Supplementary file3 (DOCX 65 kb)

Supplementary file4 (DOCX 23 kb)
