# Supplementary material for: Towards Integrated Youth Care: A Systematic Review of Facilitators and Barriers for Professionals
Source: Adm Policy Ment Health. 2020 May 18;48(1):88–105. doi: 10.1007/s10488-020-01049-8 (PMC7803720; doi:10.1007/s10488-020-01049-8)
Supplement: Supplementary file 2 — Supplementary file2 (DOCX 14 kb) [file 10488_2020_1049_MOESM2_ESM.docx]

**Appendix B. Search strategy**

The search strategy was developed in collaboration with the Walaeus Library of the Leiden University Medical Centre. The search strategy was originally developed for the PUBMED electronic database, but was adapted for other electronic databases, depending on the database and available filters.

*Pubmed Search Strategy*

 ((((("Delivery of Health Care, Integrated"[Mesh] OR " Integrated Delivery of Health Care"[tw] OR "integrated Health Care"[tw] OR "integrated HealthCare"[tw] OR "integrated Care"[tw] OR "Collaborative Care"[tw] OR "patient-centered healthcare"[tw] OR "patient-centered health care"[tw] OR "patient-centered care"[tw] OR "patient-centred healthcare"[tw] OR "patient-centred health care"[tw] OR "patient-centred care"[tw] OR "coordinated healthcare"[tw] OR "coordinated health care"[tw] OR "coordinated care"[tw] OR "co-located healthcare"[tw] OR "co-located health care"[tw] OR "co-located care"[tw] OR "colocated healthcare"[tw] OR "colocated health care"[tw] OR "colocated care"[tw] OR family centered[tw] OR family centred[tw] OR familycentered[tw] OR familycentred[tw] OR person centered[tw] OR person centred[tw] OR personcentered[tw] OR personcentred[tw] OR child centered[tw] OR child centred[tw] OR childcentered[tw] OR childcentred[tw] OR ((integrated[ti] OR integration*[ti] OR collaborative[ti] OR shared[ti] OR patient-centered[ti] OR patient-centred[ti] OR coordinated[ti] OR co-located[ti] OR colocated[ti]) AND (care[ti] OR healthcare[ti] OR "health care"[ti]))) AND ("Mental Health"[Mesh] OR mental[tw] OR "behavioral health"[tw] OR "behavioural health"[tw] OR "behavioral healthcare"[tw] OR "behavioural healthcare"[tw] OR "behavioral health care"[tw] OR "behavioural health care"[tw] OR "Psychiatry"[Mesh] OR psychiatry[tw] OR psychiatr*[tw] OR psychol*[tw] OR depression[tw] OR depressive[tw] OR "substance abuse"[tw] OR autism[tw] OR autistic[tw] OR adhd[tw] OR attention deficit[tw] OR psychotrauma*[tw] OR posttrauma*[tw] OR "post trauma"[tw] OR "post traumatic"[tw] OR intellectual disabil*[tw] OR intellectual disabl*[tw] OR mental retard*[tw] OR child protection*[tw] OR "social work"[tw] OR psychosocial[tw] OR "psycho social"[tw] OR agression[tw] OR selfinjur*[tw] OR self injur*[tw] OR oppositional behav*[tw] OR anxiety[tw] OR mood disorder*[tw] OR learning problem*[tw] OR problem behav*[tw] OR eating disorder*[tw] OR anorex*[tw] OR bulimi*[tw] OR OCD[tw] OR obsessive compuls*[tw] OR neurodevelopmental disorder*[tw] OR "neuro developmental disorder"[tw] OR "neuro developmental disorders"[tw] OR stress[tw] OR stressor*[tw] OR tic[tw] OR tics[tw] OR personality disorder*[tw] OR Substance-Related Disorder*[tw] OR addict*[tw] OR psychiatr*[all fields] OR Socioeconomic disadvantage*[tw] OR financial problem*[tw] OR youth care*[tw] OR youthcare*[tw] OR "Child Protective Services"[Mesh] OR Child Protective[tw] OR child protection*[tw] OR "Child Abuse"[Mesh] OR Abuse[tw] OR Abuses[tw] OR neglect*[tw] OR maltreat*[tw] OR primary care[tw] OR primary healthcare[tw] OR primary health care[tw] OR "Child Health Services"[Mesh] OR "Child Welfare"[Mesh] OR Child Welfare[tw] OR child care[tw] OR child health care[tw] OR child healthcare[tw] OR "Adolescent Health Services"[Mesh] OR Adolescent Health Service*[tw] OR Adolescent care[tw] OR Adolescent health care[tw] OR Adolescent healthcare[tw] OR youth health service*[tw] OR youth health care[tw] OR youth healthcare[tw]) AND ("child"[mesh] OR "Pediatrics"[MESH] OR "Neonatology"[MESH] OR "child"[tw] OR "children"[tw] OR "childhood"[tw] OR "infant"[tw] OR "infants"[tw] OR "pediatric"[tw] OR "pediatrics"[tw] OR "paediatric"[tw] OR "paediatrics"[tw] OR "baby"[tw] OR "babies"[tw] OR "toddler"[tw] OR "toddlers"[tw] OR "newborn"[tw] OR "newborns"[tw] OR "postnatal"[tw] OR "postneonatal"[tw] OR "neonatal"[tw] OR "neonate"[tw] OR "neonates"[tw] OR "suckling"[tw] OR "sucklings"[tw] OR "teen"[tw] OR "teens"[tw] OR "juvenile"[tw] OR "juveniles"[tw] OR "adolescent"[tw] OR "adolescents"[tw] OR "puberty"[tw] OR "youngster"[tw] OR "youngsters"[tw] OR "boy"[tw] OR "boys"[tw] OR "girl"[tw] OR "girls"[tw] OR "schoolchild"[tw] OR "schoolchildren"[tw] OR "stepchild"[tw] OR "stepchildren"[tw] OR youth*[tw]))) NOT ("Asia"[Mesh] OR "Africa"[Mesh] OR "South America"[Mesh] OR "Aged"[Mesh] OR "Viruses"[Mesh] OR "Palliative care"[Mesh] OR "Internal Medicine"[Mesh] OR "Respiratory Tract Diseases"[Mesh]) NOT ("Adult"[Mesh] NOT "child"[mesh]).

Filters: **Publication date from 2002/01/01 to 2018/01/01**
