# Supplementary material for: Towards Integrated Youth Care: A Systematic Review of Facilitators and Barriers for Professionals
Source: Adm Policy Ment Health. 2020 May 18;48(1):88–105. doi: 10.1007/s10488-020-01049-8 (PMC7803720; doi:10.1007/s10488-020-01049-8)
Supplement: Supplementary file 3 — Supplementary file3 (DOCX 65 kb) [file 10488_2020_1049_MOESM3_ESM.docx]

**Appendix C**

Study characteristics

| Study Number | Study | Design (method) | Setting | Principal problem or diagnosis | Respondents (n) | Intensity of integrated care | Quality |
| --- | --- | --- | --- | --- | --- | --- | --- |
| 1 | Acri et al. (2016) | Descriptive (Unclear) | Primary care | Mental health problems | YC practitioners (n=unclear) | Fully-integrated | Low |
| 2 | Adams, Hinojosa, Armstrong, Takagishi, and Dabrow (2016) | Descriptive (Questionnaire) | Primary care | Broad range of problems | YC practitioners  (n=8) | Coordinated | Medium |
| 3 | Anderson-Butcher, Lawson, and Barkdull (2002) | Descriptive (Action research) | Child Welfare | Vulnerable families | YC practitioners (n=70) | Fully-integrated | High |
| 4 | Bunik et al. (2013) | Descriptive (Questionnaire) | Primary care | Mental health problems | Policy makers and managers (n=57) | Varying | Medium |
| 5 | Burka, Van Cleve, Shafer, and Barkin (2014) | Descriptive  (Questionnaire) | Primary care;  Mental health care | Mental health problems | YC practitioners (n=30) | Fully-integrated | High |
| 6 | Callaly, von Treuer, van Hamond, and Windle (2011) | Descriptive (Focus groups and interviews) | Primary care | Mental health problems | ‘Stakeholders’  (n=unclear) | Fully-integrated | Low |
| 7 | Campo et al. (2005) | Descriptive  (Case study) | Primary care | Mental health problems | Unclear (n=unclear) | Fully-integrated | Low |
| 8 | Carbone, Behl, Azor, and Murphy (2010) | Descriptive (Focus Groups) | Mental health care | Autism Spectrum Disorder | YC practitioners  (n = 9) | Fully-integrated | High |
| 9 | Campbell et al. (2017) | Descriptive (Questionnaire) | Primary care and mental health care | Mental health problems | YC Practitioners  (n =123) | Varying | High |
| 10 | Carlson et al. (2012) | Descriptive (Questionnaire) | Early childhood system | Mental health problems, socioemotional wellbeing and children at risk of childhood expulsion | YC Practitioners  (n=unclear) | Fully-Integrated | Low |
| 11 | Collins and McCray (2012) | Descriptive (Interviews) | Social care, Education and primary care | Broad range of problems | YC Practitioners, education workers (n=20) | Coordinated | High |
| 12 | Dayton et al. (2016) | Descriptive (Unclear) | Primary care and mental health care | Trauma | YC Practitioners, parents and youth (n=unclear) | Coordinated | Low |
| 13 | Eapen and Jairam (2009) | Descriptive (Literature review) | Primary care and mental health care | Mental health problems | Unclear (n=unclear) | Unclear | Low |
| 14 | Erickson (2012) | Descriptive  (Literature review) | Juvenile Justice and mental health care | Mental health problems, criminal behavior | Unclear  (n=unclear) | Coordinated | Low |
| 15 | Fallucco et al. (2017) | Descriptive/Pilot study  (Questionnaire) | Primary care | Depression, Anxiety or ADHD | YC Practitioners  (n=25) | Linkage | High |
| 16 | Friedman et al. (2007) | Longitudinal (focus groups) | Juvenile Justice, Child care and Child protection services | Family violence | YC Practitioners  (n=varying from 25 to 51 participants in 6 different focus group rounds) | Fully integrated | High |
| 17 | Gadomski et al. (2014) | Descriptive  (Interviews) | Primary care | Mental health problems | YC Practitioners  (n=40) | Fully integrated | High |
| 18 | Gaines, Missiuna, Egan, and McLean (2008) | Descriptive (Questionnaire and focus groups) | Primary care | Developmental Coordination Disorder | YC Practitioners, occupational therapists  (n=147) | Linkage | High |
| 19 | Garcia et al. (2014) | Descriptive  (Literature review) | Child welfare, child protection services | Child maltreatment | YC Practitioners, law enforcement agencies  (n=unclear) | Varying | Low |
| 20 | Garfunkel, Pisani, leRoux, and Siegel (2011) | Quasi Experimental  (Questionnaire) | Mental health care | Mental illness | YC Practitioners  (n=147) | Fully integrated | High |
| 21 | Godoy et al. (2017) | Descriptive  (Case description) | Primary care and pediatric hospital | Mental health problems, psychosocial stressors | YC Practitioners, managers and policy makers  (n=unclear) | Fully integrated | High |
| 22 | Golding (2010) | Descriptive  (Case description, literature review) | Mental health care, education | Mental health problems | YC Practitioners,  (n=unclear) | Fully integrated | High |
| 23 | Greene, Ford, Ward-Zimmerman, Honigfeld, and Pidano (2016) | Quasi Experimental  (Survey, observation) | Mental health care and primary care | Mental health problems | YC Practitioners,  (n=39) | Coordinated | High |
| 24 | Guevara et al. (2005) | Descriptive (Focus groups) | Primary care, mental health, education | ADHD | YC Practitioners  (n=varying from 4-10 participants per focus group13 focus groups) | Linkage | High |
| 25 | Hawkins (2009) | Descriptive  (Literature Review) | Substance abuse, Mental health care | Co-occurring mental health and substance abuse disorder | YC Practitioners (n=unclear) | Varying | Medium |
| 26 | Hoffses et al. (2016) | Descriptive (Focus groups) | Primary care | Broad range of problems | YC Practitioners  (n=unclear) | Unclear | Low |
| 27 | Hyman and Johnson (2012) | Descriptive  (Literature review) | Primary care | Autism Spectrum Disorder | YC Practitioners and Parents  (n=unclear) | Fully integrated | Low |
| 28 | Hyter, Atchison, Henry, Sloane, and Black-Pond (2002) | Descriptive (Case description, literature review) | Multidisciplinary care center for trauma | Trauma | Unclear (n=unclear) | Fully integrated | Low |
| 29 | Janssens, Peremans, and Deboutte (2010) | Descriptive (Focus groups) | Mental health care | Mental health problems | YC Practitioners  (n=56) | Linkage | High |
| 30 | Kirby and Thomas (2011) | Descriptive  (Literature review) | Child and youth care services, education | Comorbid developmental disorders | Unclear (n=unclear) | Coordinated | Medium |
| 31 | Kolko et al. (2014) | Randomized controlled trial (Questionnaire) | Primary care | ADHD or Disruptive Behavior Disorder | YC Practitioners  (n=74) | Coordinated | High |
| 32 | Kolko and Perrin (2014) | Descriptive (Literature review) | Primary care | Broad range of problems | Unclear (n=unclear) | Varying | Low |
| 33 | Levy et al. (2017) | Descriptive  (Interviews) | Primary care and mental health care | Mental health problems | YC Practitioners (n=37) | Fully integrated | High |
| 34 | Liff and Andersson (2011) | Descriptive (Interviews and observations) | Mental health care | Mental health problems | YC Practitioners (n=73) | Linkage | Medium |
| 35 | Lubman, Hides, and Elkins (2008) | Descriptive  (Case description and literature review) | Alcohol and other drugs sector | Alcohol problems and co-morbid mental health problems | Unclear (n=unclear) | Fully integrated | Low |
| 36 | Lynch, Cho, Ogle, Sellman, and Dosreis (2014) | Descriptive (case study, interviews) | Primary care | ADHD | YC Practitioners (n=11) | Coordinated | High |
| 37 | Nadeau, Jaimes, Johnson-Lafleur, and Rousseau (2017) | Descriptive  (Interviews) | Primary care | Mental health problems | YC Practitioners, parents and youth (n=15) | Fully integrated | High |
| 38 | Njoroge, Williamson, Mautone, Robins, and Benton (2017) | Descriptive (Literature review) | Primary care | Broad range of problems | YC Practitioners (n=unclear) | Fully integrated | Low |
| 39 | Nolan, Walker, Hanson, and Friedman (2016) | Descriptive (Focus groups) | Primary care | Autism Spectrum Disorder | YC Practitioners  (n=25) | Coordinated | High |
| 40 | Ødegård (2006) | Validation study  (Questionnaire) | Primary care | Mental health problems | YC Practitioners (n=134) | Coordinated | High |
| 41 | Oppenheim et al. (2016) | Descriptive (Interviews, Questionnaire and case study) | Primary care | Broad range of problems | Management of YC practitioners (n=6) | Coordinated | Low |
| 42 | Reiss, Greene, and Ford (2017) | Descriptive (Interviews) | Primary care and Mental health care | Mental health problems | YC Practitioners (n=9) | Linkage | High |
| 43 | Rousseau, Pontbriand, Nadeau, and Johnson-Lafleur (2017) | Descriptive (Questionnaire) | Primary care | Mental health problems | YC Practitioners  (n=104) | Coordinated | High |
| 44 | Stuart (2012) | Descriptive  (Action research auto-ethnography) | Child care, social care, health, justice and education | Broad range of problems | YC Practitioners (n=20) | Fully integrated | Medium |
| 45 | Stuart (2014) | Descriptive Action research (interviews, observations, workgroups, system analysis) | Child care, social care, health, justice and education | Broad range of problems | YC Practitioners  (n=varying from 11-66 per cycle) | Fully integrated | High |
| 46 | Ward-Zimmerman and Cannata (2012) | Descriptive (Questionnaire) | Primary care | Mental health problems | YC Practitioners (n=46) | Coordinated | Low |
| 47 | Wayne, Alkon, and Buchanan (2008) | Descriptive  (interviews, focus groups, computer-based Delphi study) | Early care and education, mental health, parent education and family support | Broad range of problems | YC Practitioners, Policy makers, Parents and Management (focus groups n=910; interviews n=122; Delphi study n=14). | Fully integrated | High |
| 48 | Widmark, Sandahl, Piuva, and Bergman (2013) | Descriptive  (Interviews) | Child welfare | Anxiety and depression | Parents (n=7) | Linkage | High |
| 49 | Wissow, van Ginneken, Chandna, and Rahman (2016) | Descriptive (Literature Review) | Primary care and mental health care | Mental health problems | Unclear (n=unclear) | Fully integration | Low |
| 50 | Cooper, Evans, and Pybis (2016) | Descriptive (Literature review) | Mental health care | Emotional, behavioral and mental difficulties | YC Practitioners, parents, management, policy makers (n=unclear) | Varying | High |
| 51 | Davis et al. (2012) | Descriptive (Questionnaire) | Primary care | Behavioral concerns and mental health problems | YC Practitioners, (n=70) | Linkage | Medium |
| 52 | Briggs, Racine, and Chinitz (2007) | Descriptive (Case description, Questionnaire) | Infant mental health | Mental health problems or developmental problems | YC Practitioners,  (n=unclear) | Fully-Integration | Low |
| 53 | Darlington, Feeney, and Rixon (2005) | Descriptive (Interviews) | Child protection, mental health care | Parental mental health problems, youth mental health problems and child safety concerns | YC Practitioners,  (n=37) | Varying | High |
| 54 | Darlington, Feeney, and Rixton (2005) | Descriptive (Questionnaire) | Child protection, mental health care | Parental mental health problems, youth mental health problems and child safety concerns | YC Practitioners,  (n=232) | Varying | High |
| 55 | Odegard and Strype (2009) | Descriptive (Questionnaire) | Primary care, mental health care, education | Mental health problems | YC Practitioners  (n=134) | Coordinated | High |
