# Supplementary material for: Towards Integrated Youth Care: A Systematic Review of Facilitators and Barriers for Professionals
Source: Adm Policy Ment Health. 2020 May 18;48(1):88–105. doi: 10.1007/s10488-020-01049-8 (PMC7803720; doi:10.1007/s10488-020-01049-8)
Supplement: Supplementary file 4 — Supplementary file4 (DOCX 23 kb) [file 10488_2020_1049_MOESM4_ESM.docx]

Appendix D: Summary of findings table

| Theme | Subtheme (number of studies)  Study numbers | Quality ^[[1]](#footnote-1)^ | | Context | Consistency | Strength of overall evidence |
| --- | --- | --- | --- | --- | --- | --- |
| Child’s environment | Family-centered focus (n=17)  6, 11, 22, 25, 29, 30, 32, 34, 42, 44, 45, 47, 49, 50, 51, 53, 54 | Facilitators (n=12) High quality: 8 Medium quality: 1 Low quality: 3 | Barriers (n=8)  High quality: 4 Medium quality: 3  Low quality:1 | General | Mixed | Medium-Strong |
| Child’s environment | Fragmentation (n=5)  8, 11, 23, 36, 39 | Facilitators (n=0) High quality: 0 Medium quality:0  Low quality:0 | Barriers (n=5)  High quality: 5 Medium quality: 0 Low quality: 0 | General | Consistent | Strong |
| Preconditions | Time (n=25)  2, 5, 8, 10, 12, 17, 19, 21, 22, 23, 24, 27, 35, 36, 37, 39, 42, 45, 46, 47, 49, 51, 52, 54, 55. | Facilitators (n=8) High quality: 5 Medium quality: 0 Low quality: 3 | Barriers (n=19) High quality: 11 Medium quality: 2  Low quality: 6 | General | Consistent | Strong-Very strong |
| Preconditions | Financial (n=7)  2, 5, 21, 33, 39, 42, 47 | Facilitators (n=0) High quality: 0 Medium quality: 0 Low quality:0 | Barriers (n=7)  High quality: 6 Medium quality: 1 Low quality:0 | General | Consistent | Strong |
| Preconditions | Professionals and resources (n=28)  1, 2, 3, 6, 7, 8, 9, 11, 13, 17, 19, 21, 24, 27, 28, 29, 32, 33, 39, 41, 46, 48, 49, 50, 51, 52, 53, 54 | Facilitators (n=13) High quality: 6 Medium quality: 1 Low quality: 6 | Barriers (n=19)  High quality: 10 Medium quality: 2 Low quality: 7 | General | Mixed | Strong |
| Care process | Screening and assessment (n=21)  1, 8, 11, 12, 15, 17, 21, 26, 27, 28, 29, 32, 33, 35, 38, 41, 46, 49, 50, 51, 52 | Facilitators (n=19) High quality: 6 Medium quality: 1 Low quality: 12 | Barriers (n=4)  High quality: 3 Medium quality: 0 Low quality: 1 | General | Mixed | Strong |
| Care process | Shared care plan (n=5)  7, 25, 38, 39, 50 | Facilitators (n=3) High quality: 1 Medium quality: 1 Low quality: 1 | Barriers (n=2)  High quality: 1 Medium quality: 0 Low quality: 1 | General | Consistent | Medium-Strong |
| Care process | Referral (n=9)  2, 13, 24, 29, 38, 41, 50, 51, 52 | Facilitators (n=6) High quality: 1 Medium quality: 1 Low quality: 4 | Barriers (n=3)  High quality: 2 Medium quality: 1 Low quality: 0 | General | Consistent | Medium-Strong |
| Expertise | Knowledge and training (n=37)  1, 2, 3, 4, 5, 10, 11, 12, 13, 14, 15, 16, 17, 18, 19, 20, 21, 24, 25, 26, 27, 29, 30, 32, 33, 35, 38, 39, 41, 44, 46, 49, 50, 51, 52, 53, 54 | Facilitators (n=30):  High quality: 11 Medium quality: 4 Low quality: 15 | Barriers (n=17):  High quality: 9 Medium quality: 4 Low quality: 4 | General | Mixed | Strong |
| Expertise | Guidelines (n=13)  3, 7, 8, 19, 23, 25, 27, 30, 37, 38, 39, 42, 50 | Facilitators (n=11) High quality: 7 Medium quality: 1 Low quality: 3 | Barriers (n=3)  High quality: 1 Medium quality: 1 Low quality: 1 | General | Consistent | Strong |
| Expertise | Self-efficacy (n=15)  8, 9, 15, 17, 20, 24, 27, 30, 33, 35, 39, 45, 49, 51, 53 | Facilitators (n=6) High quality: 4  Medium quality: 1 Low quality: 1 | Barriers (n=12)  High quality: 8 Medium quality: 1 Low quality:3 | General | Consistent | Strong |
| Interprofessional collaboration | General aspects of interprofessional collaboration (n=10)  3, 19, 22, 29, 34, 37, 39, 40, 42, 45. | Facilitators (n=8) High quality: 7 Medium quality: 0  Low quality: 1 | Barriers (n=3)  High quality: 2 Medium quality: 1 Low quality: 0 | General | Mixed | Medium-Strong |
| Interprofessional collaboration | Familiarity with other professionals (n=16)  3, 6, 11, 12, 14, 22, 23, 29, 32, 33, 37, 42, 45, 46, 50, 53 | Facilitators (n=14) High quality: 9  Medium quality: 0 Low quality: 5 | Barriers (n=6)  High quality: 6 Medium quality: 0 Low quality: 0 | General | Consistent | Strong-Very Strong |
| Interprofessional collaboration | Forms: Co-location (n=19)  4, 6, 12, 15, 16, 19, 21, 23, 29, 30, 31, 33, 37, 39, 43, 46, 47, 50, 52. | Facilitators (n=18) High quality: 11 Medium quality: 2 Low quality:5 | Barriers (n=2)  High quality: 2 Medium quality: 0 Low quality:0 | General | Mixed | Strong |
| Interprofessional collaboration | Forms: Multidisciplinary meetings (n=13)  10, 16, 19, 21, 22, 28, 29, 33, 41, 42, 48, 50, 53 | Facilitators (n=13) High quality: 9 Medium quality: 0 Low quality: 4 | Barriers (n=2)  High quality: 2 Medium quality: 0  Low quality: 0 | General | Consistent | Strong-Very Strong |
| Interprofessional collaboration | Forms: Consultation (n=18)  1, 7, 10, 12, 15, 17, 20, 22, 24, 29, 32, 35, 38, 41, 50, 51, 52, 54 | Facilitators (n=13) High quality: 5 Medium quality: 0 Low quality: 8 | Barriers (n=9)  High quality: 7 Medium quality: 1  Low quality:1 | General | Consistent | Strong |
| Interprofessional collaboration | Forms: Care-coordination (n=6)  7, 10, 29, 42, 50, 55 | Facilitators (n=6) High quality: 4 Medium quality: 0  Low quality: 2 | Barriers (n=0)  High quality: 0 Medium quality: 0 Low quality: 0 | General | Consistent | Medium |
| Information exchange | Communication (n=23)  6, 9, 11, 12, 23, 24, 25, 26, 27, 30, 32, 34, 36, 37, 38, 39, 42, 44, 45, 46, 48, 50, 53. | Facilitators (n=19) High quality: 10 Medium quality: 2 Low quality: 7 | Barriers (n=9)  High quality: 8 Medium quality: 1 Low quality: 0 | General | Consistent | Strong-Very Strong |
| Information exchange | Sharing information/ confidentiality (n=27)  9, 12, 14, 16, 19, 21, 23, 26, 27, 28, 29, 30, 32, 33, 34, 36, 37, 38, 41, 42, 46, 47, 48, 50, 51, 53, 54 | Facilitators (n=20) High quality: 8 Medium quality: 2 Low quality:10 | Barriers (n=14)  High quality: 11 Medium quality: 1 Low quality: 2 | General | Mixed | Strong |
| Professional identity | Professional roles and responsibilities (n=27)  6, 11, 14, 19, 21, 22, 23, 24, 26, 29, 30, 33, 34, 36, 37, 38, 39, 42, 44, 45, 48, 50, 51, 53, 54, 55. | Facilitators (n=14) High quality: 7 Medium quality: 2 Low quality: 5 | Barriers: (n=17)  High quality: 14 Medium quality: 2 Low quality: 1 | General | Mixed | Strong |
| Professional identity | Attitudes (n=16)  4, 12, 14, 17, 19, 22, 23, 24, 29, 33, 34, 42, 44, 45, 54, 55 | Facilitators (n=11) High quality: 8 Medium quality: 1  Low quality: 2 | Barriers: (n=10)  High quality: 6  Medium quality: 2  Low quality: 2 | General | Consistent | Strong |
| Professional identity | Shared thinking (n=22)  3, 6, 9, 11, 12, 14, 19, 25, 26, 30, 34, 37, 38, 40, 42, 45, 47, 50, 52, 53, 54, 55. | Facilitators (n=10) High quality: 5 Medium quality: 1 Low quality: 4 | Barriers (n=15)  High quality: 9 Medium quality: 2 Low quality: 4 | General | Consistent | Strong-Very Strong |
| Professional identity | Trust, respect and equality (n=20)  6, 11, 16, 19, 24, 26, 29, 33, 34, 35, 37, 38, 40, 42, 44, 45, 47, 48, 50, 54 | Facilitators (n=12) High quality: 6 Medium quality: 1 Low quality: 5 | Barriers (n=13)  High quality: 10 Medium quality: 2  Low quality: 1 | General | Consistent | Strong |

1. Based on critical appraisal of individual studies. [↑](#footnote-ref-1)
